# Supplementary material for: Super-Resolution Microscopy Analysis of Hepatitis B Viral cccDNA and Host Factors
Source: Viruses. 2023 May 16;15(5):1178. doi: 10.3390/v15051178 (PMC10223333; doi:10.3390/v15051178)
Supplement: Supplementary file 1 [file viruses-15-01178-s001.zip › viruses-2379421-supplementary.pdf]

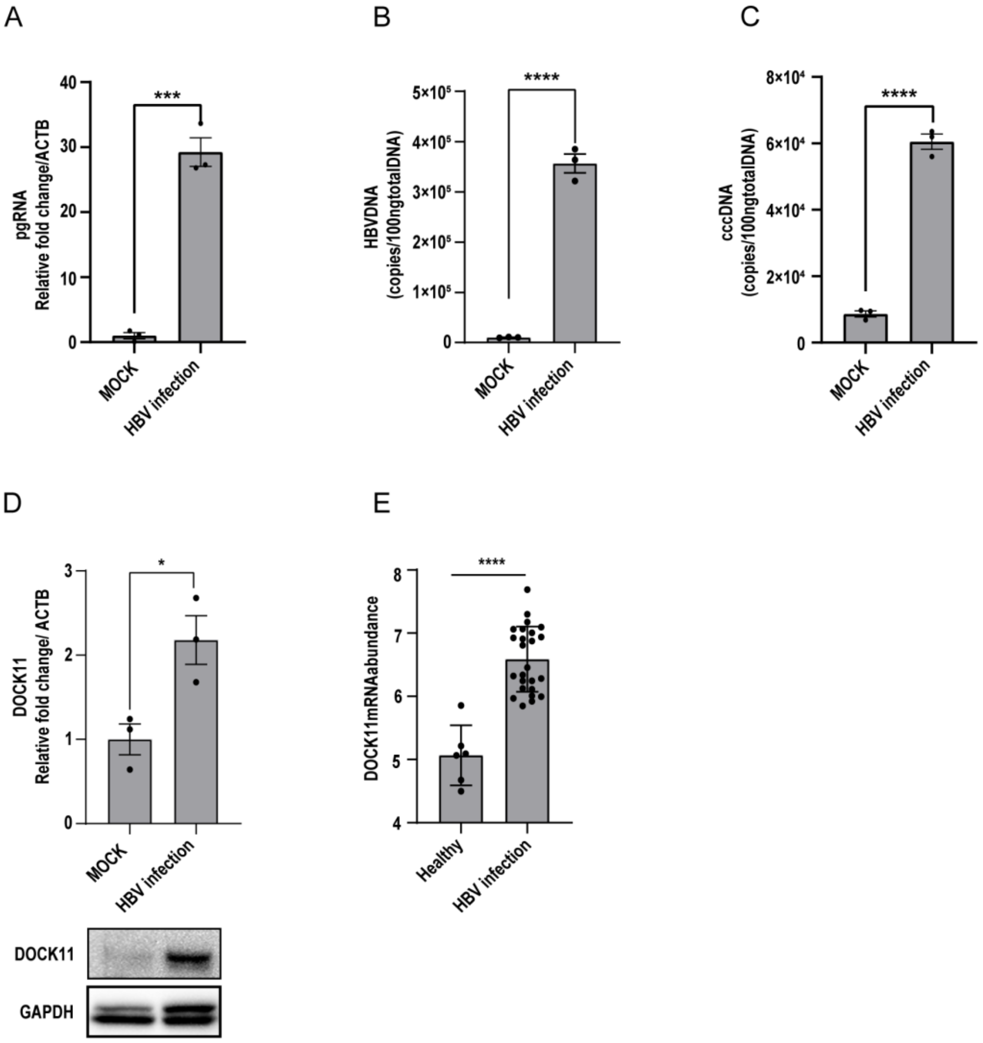

Figure S1. Effect of HBV infection on the expression of DOCK11. HepG2-hNTCP C4 cells were infected with HBV particles from HepAD38 cells at a MOI of 1000 GEq/cell. At 1 week post-infection, the cells were collected to extract DNA and RNA for PCR reactions. HBV DNA (A), pgRNA (B), and cccDNA (C) from cell lysates of HBV-infected cells were measured by real-time PCR. DOCK11 expression (D) was detected by both western blot and real-time PCR. (B) The expression of DOCK11 was analyzed in HBV-infected and uninfected human liver tissue from a published dataset (GEO: GSE83148). *p < 0.05, **p < 0.01, ***p < 0.001, ****p < 0.0001.

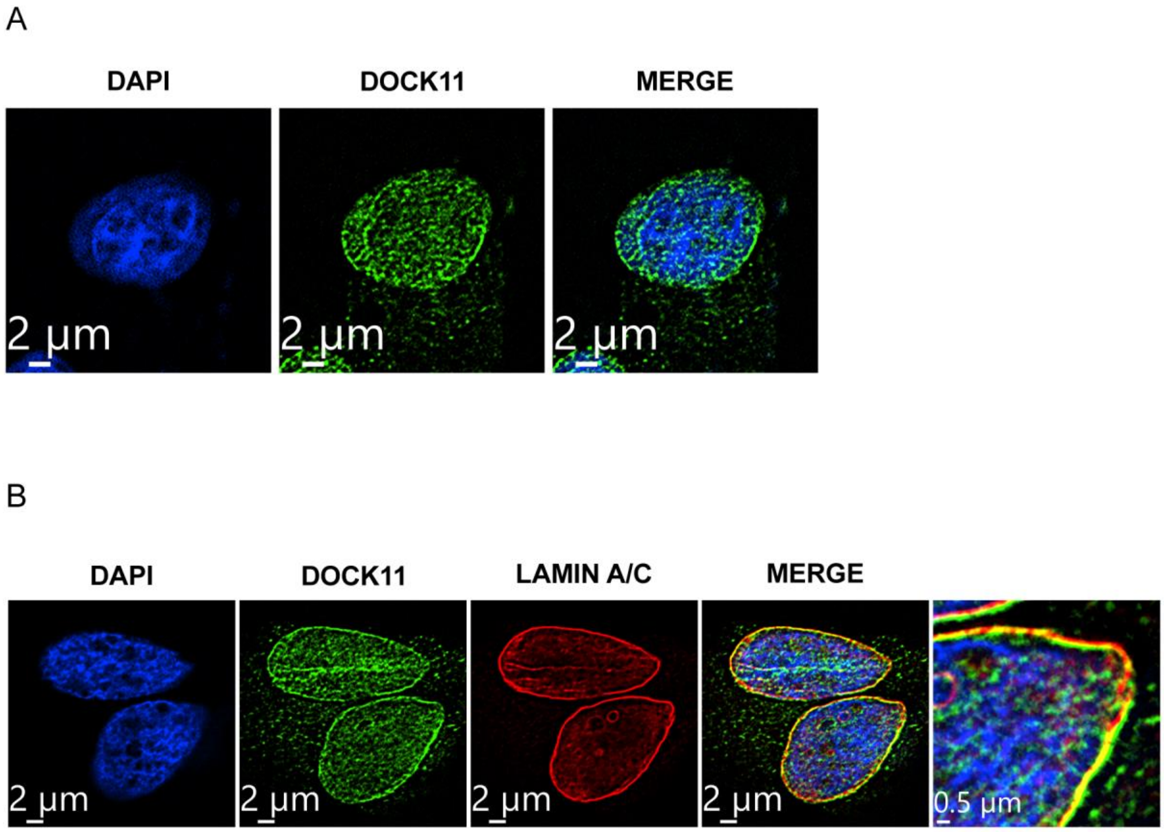

Figure S2. Localization of DOCK11. HepAD38 cells were stained with DOCK11 antibody (green) (A) and lamin A/C antibody (red) (B). The subcellular localizations of DOCK11 and lamin A/C were determined by Dragonfly confocal microscopy using a 60× objective. Nuclei were stained with DAPI (blue). Scale bars, 2 μm and 0.5 μm.

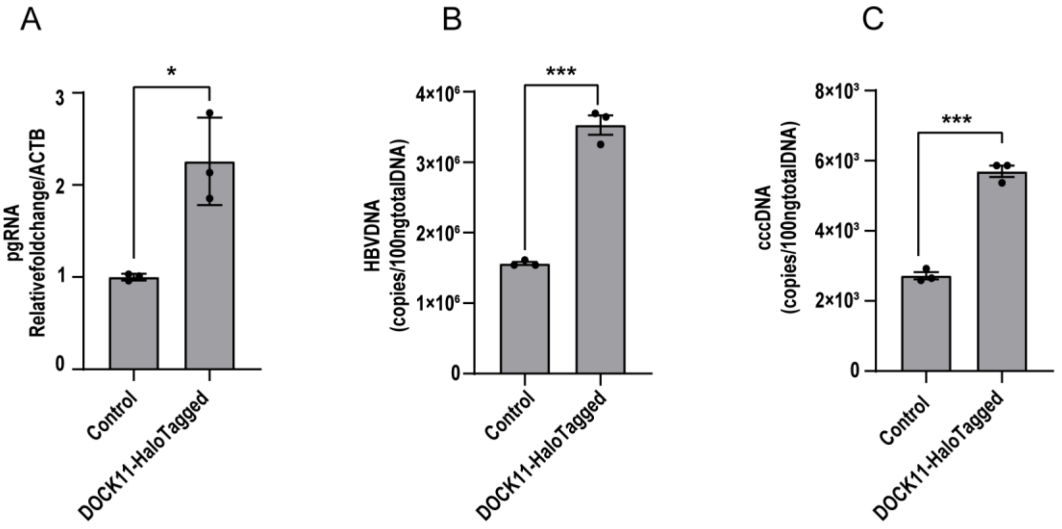

Figure S3. Overexpression of DOCK11 promotes HBV transcription and replication. HepAD38 cells were transiently transfected with Halo-DOCK11 vector or empty vector. After transfection, the cells were collected to extract DNA and RNA for PCR reactions. HBV DNA (A), pgRNA (B), and cccDNA (C) from cell lysates were measured by real-time PCR. *p < 0.05, **p < 0.01, ***p < 0.001, ****p < 0.0001.

Table S1. Key Resources

	Antibody	Company	Cat. No.	Application
1	H3K4me3 monoclonal antibody	Thermo Fisher Scientific	MA5-11199	IF, ChIP, WB
2	H3K27me3 antibody	Thermo Fisher Scientific	MA5-11198	IF, ChIP, WB
4	DOCK11	Bethyl	A301-638A	WB
5	Anti-HP1α [EPR5777] (heterochromatin marker)	Abcam	ab109028	WB, ChIP
6	Anti-HP1α [EPR5777] Alexa Fluor® 647 (heterochromatin marker)	Abcam	ab198391	IF
7	Phospho-RNA pol II CTD (Ser2) Recombinant Rabbit Monoclonal Antibody (7H23L1)	Thermo Fisher Scientific	703108	IF, ChIP, WB
9	β-Actin antibody	Cell Signaling Technology	4967	WB
10	Anti-GAPDH antibody [EPR16891] (loading control)	Abcam	ab181602	WB
11	Anti-Histone H3 antibody (nuclear marker) and ChIP Grade	Abcam	ab1791	WB

Table S2. Oligonucleotides for RT-PCR

PCR probe set	Primer	Sequence (5′–3′)
HBV DNA	Forward	5′-ACTCACCAACCTCCTGTCCT-3′
	Reverse	5′-GACAAACGGGCAACATACCT-3′
	Probe	5′-FAM-TATCGCTGGATGTGTCTGCGGCGT-TAMRA-3′
cccDNA	Forward	5′-CGTCTGTGCCTTCTCATCTGC-3′
	Reverse	5′-GCACAGCTTGGAGGCTTGAA-3′
	Probe	5′-FAM-CTGTAGGCATAAATTGGT-MGB-3′
pgRNA	Forward	5′-GCTCTGTATCGGGAGGCCTTA-3′
	Reverse	5′-TGAGTGCTGTATGGTGAGGAGAA-3′
	Probe	5′-FAM-AGTCTCCGGAACATT-MGB-3′
